# Effects of Renal Denervation via Renal Artery Adventitial Cryoablation on Atrial Fibrillation and Cardiac Neural Remodeling

**DOI:** 10.1155/2018/2603025

**Published:** 2018-12-11

**Authors:** Wei Wang, Zhaolei Jiang, Rongxin Lu, Hao Liu, Nan Ma, Jie Cai, Min Tang, Ju Mei

**Affiliations:** Department of Cardiothoracic Surgery, Xinhua Hospital, Shanghai Jiaotong University School of Medicine, Shanghai 200092, China

## Abstract

**Introduction:**

Catheter-based renal denervation (RDN) could reduce cardiac sympathetic nerve activity (SNA) and inhibit atrial fibrillation (AF). However, the reliability is uncertain, because the renal sympathetic nerves are mainly distributed in the adventitial surface of the renal artery.

**Objective:**

The aims of this study were to test the hypothesis that renal artery adventitial ablation (RAAA) definitely had the effects of RDN and to study the effects of RDN via renal artery adventitial cryoablation (RAAC) on AF and cardiac neural remodeling.

**Methods:**

Twenty beagle canines were randomly assigned to two groups: the left RDN group (LRDN, *n*=10), which underwent left RDN via RAAC; the Sham group (*n*=10). After 2 months of postoperative recovery, AF vulnerability, AF duration, and histological examination were performed in both groups.

**Results:**

Compared with the Sham group, left stellate ganglion (LSG) tissue fibrosis was increased in the LRDN group. LRDN significantly increased the percentage of TH-negative ganglionic cells and decreased the density of TH-positive nerves in the LSG (*P* < 0.001). Also, the densities of TH-positive nerves and GAP43 immunoreactivity within the left atrium (LA) were significantly decreased in the LRDN group (*P* < 0.05). After LA burst pacing, all 10 canines (100%) could be induced AF in the Sham group, but only 4 of 10 canines (40%) could be induced AF in the LRDN group (*P*=0.011). The percentage of LA burst stimulation with induced AF was 26.7% (8/30) in the LRDN group, which was significantly decreased compared with that of the Sham group (53.3%, 16/30) (*P*=0.035). In addition, AF duration was also significantly decreased in the LRDN group (13.3 ± 5.1 s) compared with that of the Sham group (20.3 ± 7.3 s, *P*=0.024).

**Conclusions:**

RDN via RAAC could cause cardiac neural remodeling and effectively inhibit AF inducibility and shorten AF duration. It may be useful in selecting therapeutic approaches for AF patients.

## 1. Introduction

Atrial fibrillation (AF) is the most common cardiac arrhythmia [1]. Autonomic neural system (ANS) activation played a key role in the occurrence and maintenance of AF, which could induce atrial structural remodeling and the changes of atrial electrophysiology. Reasonable autonomic nerve intervention could reduce cardiac sympathetic nerve activity (SNA) and improve the treatment of AF [2–4]. Several studies have showed that catheter-based renal denervation (RDN) could reduce cardiac SNA and inhibit AF [5–7]. Linz et al. [5] demonstrated that catheter-based RDN could reduce atrial sympathetic nerve sprouting, structural alterations, and AF complexity in goats with persistent AF. Wang et al. [6] found that catheter-based RDN could inhibit the progression of paroxysmal AF by reducing the incidences of AF and shortening the duration of AF. However, the reliability of catheter-based RDN is uncertain, because the renal sympathetic nerves are mainly distributed in the adventitial surface of the renal artery [8, 9]. We speculated that renal artery adventitial ablation (RAAA) may have better effects of RDN than catheter-based RDN. At present, epicardial cryoablation has been widely used in the surgical AF ablation, which could achieve satisfactory integrity and transmurality of ablation lines. Therefore, we aimed to perform this study as following: (1) to test the hypothesis that RAAA definitely had the effects of RDN; (2) to study the effects of RDN via renal artery adventitial cryoablation (RAAC) on AF and cardiac neural remodeling.

## 2. Methods

### 2.1. Animals

This study was approved by the Ethics Committee of Xinhua Hospital, Shanghai Jiao Tong University, School of Medicine. All animal experiments were performed in accordance with the National Institutes of Health Guide for the Care and Use of Laboratory Animals. A total of 20 beagle canines (male, 15–20 kg) were studied in the Experimental Animal Center of Xinhua Hospital. The 20 beagle canines were randomly assigned to the following two groups: (1) the left RDN group (LRDN, *n*=10), which underwent left RDN via RAAC; (2) the Sham group (*n*=10).

### 2.2. Renal Artery Adventitial Ablation (RAAA)

**Figure 1:**
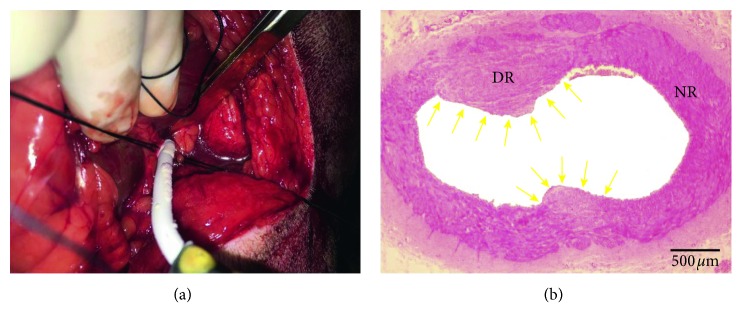
Renal artery ablation. (a) Renal artery adventitial cryoablation (RAAC). (b) Morphological changes of the LRA after LRDN. DR: damage region; NR: normal region.

Routine clinical monitoring was performed during the surgical procedure. Anesthesia was induced with ketamine (5–10 mg/kg) and midazolam (0.1–0.2 mg/kg IV). After intubation and mechanical ventilation, anesthesia was maintained with 2.0% isoflurane. The canines were placed in the right lateral decubitus position. A 6 to 8 cm subcostal incision was made on the abdomen to separate and expose the left renal artery (LRA). The proximal renal artery close to the renal artery ostium was subjected to cryoablation (both ventral side and dorsal side of the renal artery, cryoablation temperature −70°C, 120 seconds for each side) using a cryoprobe (5 cm in length, with a diameter of 6 mm on the tip, CryoICE, Atricure, USA) (Figure 1(a)). The incision was then closed. Cefuroxime sodium (30 mg/kg) was administered by intravenous perfusion during the surgery and used for three days after the surgery.

RAAC was performed for all canines in the LRDN group. For the Sham group, we only separated and exposed the LRA through the same incision, but RAAC was not performed.

### 2.3. AF Vulnerability Studies

After 2 months of postoperative recovery, electrophysiology experiment was performed. After the canines were anesthetized, the tips of two pairs of looping electrodes were directly sutured to the surface of the left atrial appendage (LAA) through the left third intercostal incision. One pair of looping electrodes was used to record the left atrial (LA) electric signal, and the other pair of looping electrodes was used for pacing. The electrocardiograms and electric signals were recorded simultaneously using the LabChart system (ADInstruments, AUS). Continuous 6 hours' rapid atrial pacing (RAP) (600 bpm, 0.5 ms, twice threshold current) was administered at the LA site for all canines. After that, AF inducibility was assessed by the burst pacing protocol at the LA site. Burst pacing was performed for each canine, at a cycle length of 50 ms and a stimulus output of 0.5 V plus twice threshold current for 60 s. Signals were sampled at 2 kHz and stored with the LabChart system. AF was defined as irregular atrial rates >500 bpm and lasting over 5 s associated with irregular atrioventricular conduction. AF inducibility was repeated for 3 times by burst pacing.

### 2.4. Histologic Analysis

After the electrophysiology experiment, the canines were then euthanized, and the tissues of LRA, left stellate ganglion (LSG), and LA were harvested. Small portions of the tissues were fixed in 4% formalin for 45 min and then stored in 70% alcohol for analysis. The tissues were paraffin embedded and cut into 5 *μ*m thick sections routinely. The LRA was stained with haematoxylin-eosin (HE). Immunohistochemical staining of the LSG was performed using an anti-tyrosine hydroxylase- (TH-) antibody (22941, Immunostar, USA). Immunohistochemical staining of the LA was performed using an anti-TH-antibody (22941, Immunostar, USA) and an anti-growth-associated protein 43- (GAP-43-) antibody (NB300-143SS, Novus, USA). LSG tissues were also stained with the Masson trichrome stain. All slides were examined manually under a DP72 microscope (Olympus, Tokyo, Japan).

### 2.5. Statistical Analysis

The software SPSS 22.0 (SPSS, USA) was used for the statistical analysis. Continuous variables were expressed as mean ± standard deviation (SD) and 95% confidence interval (CI). Student's *t* test was used to compare continuous variables between the two groups. Categorical variables were presented as frequencies and proportions. The chi-square test or Fisher's exact test was used to compare categorical variables. A *P* value of <0.05 was considered to be significant for these comparisons.

## 3. Results

### 3.1. Effects of LRDN on the Morphological Changes of LRA

After 2 months of postoperative recovery, the canines were sacrificed and LRA was harvested.  Figure 1(b) shows the morphological features of the LRA with H&E staining at 2 months after RAAC. The adventitia, media, and intima of renal artery wall were injured by RAAC, which displayed as the damaged region (DR). Compared with the normal region (NR), neointima formation (yellow arrow head) could be found corresponding to the DR. These features of the LRA demonstrated that RAAC could create transmural ablation from the adventitia to the intima of renal artery.

### 3.2. Effects of LRDN on Neural Remodeling of LSG

**Figure 2:**
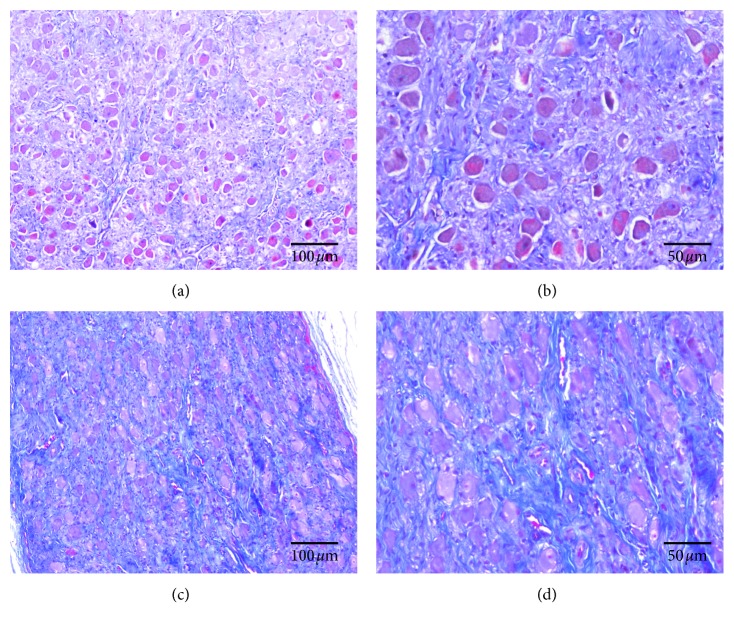
Masson trichrome staining of LSG. (a) and (b): Tissue fibrosis seen at low magnification in Sham group; (c) and (d): Tissue fibrosis seen at high magnification in LRDN group.

Figure 2 shows typical examples of LSG structural remodeling in both the Sham group and the LRDN group. Compared with the Sham group (Figures 2(a) and 2(b)), LSG tissue fibrosis was increased in the LRDN group (Figures 2(c) and 2(d)).

**Figure 3:**
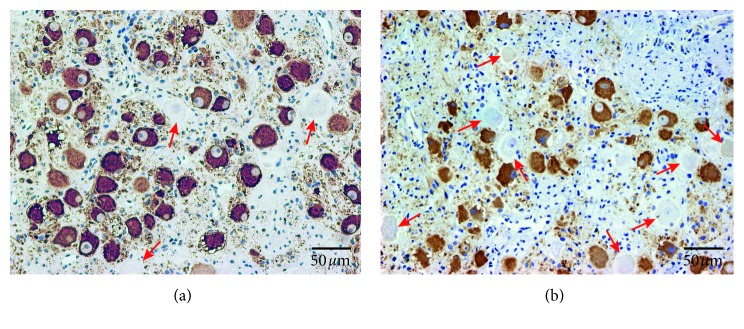
Tyrosine hydroxylase (TH) immunostaining of LSG. (a) TH staining showed TH-negative ganglion cells (red arrows) and TH-positive ganglion cells in LSG in Sham group; (b) TH staining showed TH-negative ganglion cells (red arrows) and TH-positive ganglion cells in LSG in LRDN group.

**Figure 4:**
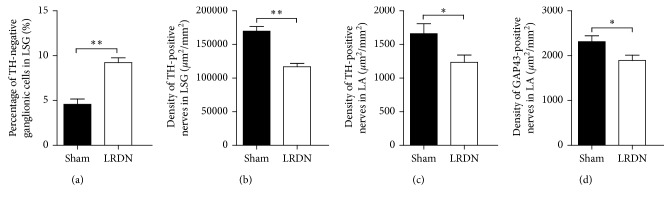
Immunostaining results in LSG or LA between Sham group and LRDN group. (a) The percentage of TH-negative ganglionic cells in LSG in both Sham group and LRDN group; (b) comparison between the density of TH-positive nerves in LSG between Sham group and LRDN group; (c) the density of TH-positive nerves in LA in both Sham group and LRDN group; (d) comparison between the density of GAP43-positive nerves in LA between Sham group and LRDN group (^*∗*^*P* < 0.05 vs. the Sham group; ^*∗∗*^*P* < 0.01 vs. the Sham group).

Figure 3 shows TH staining of the LSG in both the Sham group (Figure 3(a)) and the LRDN group (Figure 3(b)). Compared with that of the Sham group, LRDN significantly increased the percentage of TH-negative ganglionic cells in the LSG in canines of the LRDN group. The mean percentage of TH-negative ganglionic cells in canines of the LRDN group (9.2 ± 1.7% (95% CI, 8.2% to 10.3%)) was significantly higher than that in the Sham group (4.6 ± 1.9% (95% CI, 3.4% to 5.8%)) (*P* < 0.001, Figure 4(a)). For canines with LRDN, there was a significantly decreased density of TH-positive nerves in the LSG (116835.8 ± 15794.8 *µ*m^2^/mm^2^ (95% CI, 106510.7 to 125826.5)) compared to the canines of the Sham group (169784.5 ± 21284.6 *µ*m^2^/mm^2^ (95% CI, 156184.9 to 182772.4)) (*P* < 0.001, Figure 4(b)).

### 3.3. Effects of LRDN on Neural Remodeling of LA

**Figure 5:**
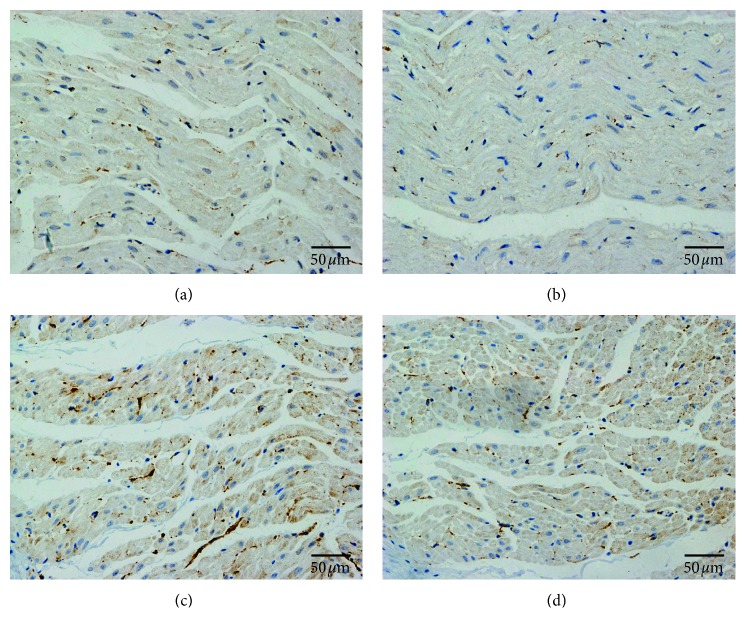
TH and GAP43 immunostaining of LA. (a) TH staining showed the densities of TH-positive nerves (brown) within the LA in Sham group; (b) TH staining showed the densities of TH-positive nerves within the LA in LRDN group; (c) GAP43 staining showed the density of GAP43 immunoreactivity in the LA in Sham group; (d) GAP43 staining showed the density of GAP43 immunoreactivity in the LA in LRDN group.

Figure 5 compares the results of immunostaining of TH and GAP43 in the LA between the Sham group and the LRDN group. The nerve density of LA for each group was expressed as a mean of nerve densities. Compared with the Sham group (Figure 5(a), 1660.6 ± 468.4 *µ*m^2^/mm^2^ (95% CI, 1385.5 to 1958.3)), the densities of TH-positive nerves within the LA were significantly decreased in the LRDN group (Figure 5(b), 1233.4 ± 345.4 *µ*m^2^/mm^2^ (95% CI, 1032.4 to 1455.7)) (*P*=0.032, Figure 4(c)). The density of GAP43 immunoreactivity in the LA was (2313.9 ± 411.0 *µ*m^2^/mm^2^ (95% CI, 2048.4 to 2560.6)) in the Sham group (Figure 5(c)), which was significantly higher than that of the LRDN group (Figure 5(d), 1890.9 ± 383.8 *µ*m^2^/mm^2^ (95% CI, 1648.9 to 2115.1)) (*P*=0.029, Figure 4(d)).

### 3.4. Effects of LRDN on AF Inducibility and AF Duration

**Figure 6:**
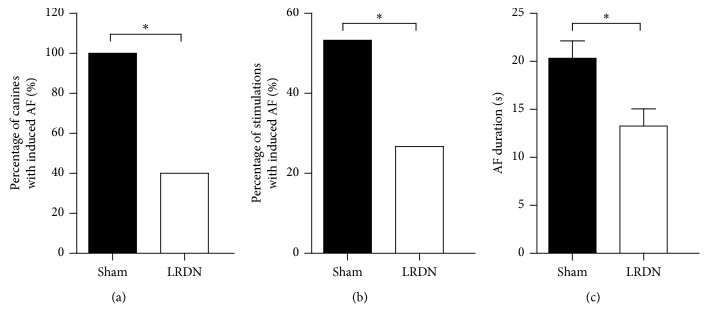
AF inducibility and AF duration between Sham group and LRDN group. (a) The percentage of canines with induced AF in LSG in both Sham group and LRDN group; (b) comparison between the percentage of stimulations with induced AF between Sham group and LRDN group; (c) comparison between the AF duration between Sham group and LRDN group (^*∗*^*P* < 0.05 vs. the Sham group).

After LA burst pacing, all 10 canines (100%) could be induced AF in the Sham group, but only 4 of 10 canines (40%) could be induced AF in the LRDN group (*P*=0.011, Figure 6(a)). The percentage of LA burst stimulation with induced AF was 26.7% (8/30) in the LRDN group, which was significantly decreased compared with that of the Sham group (53.3%, 16/30) (*P*=0.035, Figure 6(b)). In addition, AF duration was also significantly decreased in the LRDN group (13.3 ± 5.1 s, 95% CI 10.2∼17.2) compared with that of the Sham group (20.3 ± 7.3 s, 95% CI 16.9∼24.0, *P*=0.024, Figure 6(c)).

## 4. Discussion

ANS activation could induce significant and heterogeneous changes of atrial electrophysiology. Multiple evidences have demonstrated that ANS, especially sympathetic nervous system, may play a key role in the occurrence and maintenance of AF [10, 11]. In anatomical structure, the heart is innervated by the extrinsic nervous system and the intrinsic nervous system. Both the extrinsic and intrinsic cardiac nervous systems are important for arrhythmogenesis, such as AF [2, 12]. The extrinsic cardiac nervous system mainly includes stellate ganglion (SG) and vagal nerve. The intrinsic cardiac nerves are mostly found in the atrial wall. Neuromodulation methods that reduce sympathetic nerve activity may be helpful in controlling AF [13, 14]. In the previous study, Shen et al. has reported that vagal nerve stimulation (VNS) could effectively suppress SGNA and reduce the incidences of paroxysmal atrial tachyarrhythmias (PAT) [15]. Therefore, the modulation of ANS may be a promising target for intervention in AF patients. At present, ganglionated plexi (GP) ablation has been used to cure AF in patients [16–18]. However, nerve regeneration after GP ablation is still related to AF recurrence [19, 20].

In order to improve the effects of ANS modulation, several methods have been developed. RDN was considered to be one of the recommended methods to modulate ANS. Catheter-based approach has been developed for RDN, which has been proved to be able to reduce cardiac SNA and inhibit AF [21]. However, the outcomes of catheter-based RDN are still uncertain, because the afferent and efferent nerves are mainly distributed in the adventitia of the renal artery [8, 9]. In this study, we performed RDN via RAAC (both ventral side and dorsal side of the renal artery, cryoablation temperature −70°C, 120 seconds for each side). LRA with H&E staining showed that the adventitia, media, and intima of renal artery wall were injured by RAAC, which demonstrated that RAAC could create transmural ablation of renal artery for RDN.

In the previous studies, Huang et al. demonstrated that 3 hours of left-sided electrical stimulation of renal sympathetic nerve was able to increase both systemic and cardiac sympathetic nerve activities and cause neural remodeling in the LSG, which can be proarrhythmic in dogs in the presence of acute myocardial infarction. It suggested that there were direct or indirect connections between renal sympathetic nerve and LSG [22, 23]. Tsai et al. founded that RDN could reduce SG nerve activity and cause sympathetic nerve remodeling. His findings in part certificated the connections between renal sympathetic nerve and LSG [24]. According to these previous reports, increasing renal SNA could increase SG nerve activity and cause corresponding SG neural remodeling, whereas decreasing renal SNA could decrease SG nerve activity and cause corresponding SG neural remodeling. In our study, we also found that RDN via RAAC could increase LSG tissue fibrosis, increase the percentage of TH-negative ganglionic cells, and significantly decrease the density of TH-positive nerves in the LSG. That is, sympathetic components in LSG were significantly reduced by RDN via RAAC. Therefore, our findings demonstrated that RAAC definitely had the effects of RDN and inhibited SG nerve activity.

SG is the sympathetic ganglion formed by fusion of the inferior cervical ganglion and the first thoracic ganglion. SG is considered to be an important source of cardiac sympathetic innervation. It gave rise to sympathetic nerves that innervate atrium and ventricle [25, 26]. Cao et al. induced cardiac sympathetic nerve sprouting by infusing nerve growth factor (NGF) to the LSG in dogs with myocardial infarction and complete atrioventricular block [27]. This demonstrated that neural remodeling of intrinsic cardiac nerve was related to the extrinsic cardiac nerve. In our study, we harvested the LA tissues and examined with immunostaining of TH and GAP43. Both the density of TH-positive nerve and GAP43 immunoreactivity within the LA were significantly decreased in the LRDN group compared with that of the Sham group. Our findings demonstrated that LRDN not only significantly decreased the components of sympathetic nerve but also significantly inhibited novel sympathetic nerve sprouting in LA.

Overactivity of the sympathetic nervous system played an important role in the occurrence and maintenance of AF. Increased SNA or atrial sympathetic innervation is associated with increased incidence and duration of AF. In chronic AF patients, atrial sympathetic nerve densities are also significantly increased [28, 29]. Now, it was suggested that RDN could reduce SNA and decrease susceptibility to AF [5–7]. In our study, we also examined the effects of LRDN on AF inducibility and AF duration. Compared with the Sham group (100%), only 40% canines could be induced AF by LA burst pacing in the LRDN group. The percentage of LA burst stimulation with induced AF was 26.7% in the LRDN group, which was significantly lower than that of the Sham group (53.3%). Besides, LRDN also significantly decreased AF duration. Our data confirmed that RDN via RAAC could effectively inhibit AF inducibility and shorten AF duration.

## 5. Clinic Implications

The present study showed that RAAC could achieve the effects of RDN and inhibit cardiac SNA. Compared with the Sham group, RDN via RAAC could effectively inhibit AF inducibility and shorten AF duration. Therefore, we conclude that additional RDN via RAAC may be able to inhibit the occurrence and maintenance of AF and improve the therapeutic effects of AF.

## 6. Limitations

The present study has few limitations. Firstly, we did not evaluate the SG function by directly recording SG nerve activity, which may reflect SG function more objectively. Secondly, we only performed left side RDN. The reason is that previous studies [22, 23] have demonstrated that left-sided electrical stimulation of renal sympathetic nerve was able to increase both systemic and cardiac sympathetic activity and cause neural remodeling in LSG. We guess that LRDN may be able to decrease systemic and cardiac SNA. So, we would like to examine the effects of LRDN by this study. Thirdly, we got satisfactory short-term effects of RDN, but the midterm or long-term effects were not observed. The midterm or long-term effects of RDN via RAAC remain to be explored in the future. Fourthly, RDN via adventitial ablation may be potentially better than catheter-based RDN, but RDN via adventitial ablation have more trauma than that of catheter-based RDN.

## 7. Conclusions

Renal artery adventitial ablation (RAAA) definitely had the effects of RDN. RDN via renal artery adventitial cryoablation (RAAC) could cause cardiac neural remodeling and effectively inhibit AF inducibility and shorten AF duration. It may be useful in selecting therapeutic approaches for AF patients.

## Data Availability

The data used to support the findings of this study are available from the corresponding author upon request.
